# Encephalopathic Susac’s Syndrome associated with livedo racemosa in a young woman before the completion of family planning

**DOI:** 10.1186/1471-2377-13-185

**Published:** 2013-11-25

**Authors:** Maik Engeholm, Beate Leo-Kottler, Hansjörg Rempp, Tobias Lindig, Holger Lerche, Ilka Kleffner, Melanie Henes, Marcel Dihné

**Affiliations:** 1 Department of Neurology and Hertie Institute for Clinical Brain Research, Hoppe-Seyler-Str. 3, 72076 Tübingen, Germany; 2 Center for Ophthalmology, Schleichstr. 12, 72076 Tübingen, Germany; 3 Department of Diagnostic and Interventional Radiology, Hoppe-Seyler-Str. 3, 72076 Tübingen, Germany; 4 Department of Diagnostic and Interventional Neuroradiology, Hoppe-Seyler-Str. 3, 72076 Tübingen, Germany; 5 Department of Neurology, Albert-Schweitzer-Campus 1, 48149 Münster, Germany; 6 Department of Gynecology and Obstetrics, Calwerstr. 7, 72076 Tübingen, Germany

## Abstract

**Background:**

Susac’s Syndrome (SS) consists of the triad of encephalopathy, branch retinal artery occlusions (BRAO) and hearing loss (HL). Histopathologically, SS is characterised by a microangiopathy, and some observations suggest that an immune-mediated damage of endothelial cells might play a role. These findings also implicate a similarity between SS and other autoimmune diseases, most notably juvenile dermatomyositis (JDM). However, SS and JDM are commonly thought to affect distinct and non-overlapping sets of organs, and it is currently not clear how these specificities arise. Moreover, in the absence of clinical trials, some authors suggest that therapeutic approaches in SS should rely on the model of other autoimmune diseases such as JDM.

**Case presentation:**

Here, we report a case of SS in a 32-year-old pregnant woman. She initially was admitted to the hospital with subacute severe encephalopathy and multifocal neurologic signs. As cranial magnetic resonance imaging (MRI) revealed multifocal white matter lesions including the corpus callosum, erroneously a diagnosis of multiple sclerosis (MS) was made, and intravenous methylprednisolone (IVMP) therapy was initiated. A few days later, an exanthema appeared on the trunk and extremities, which was diagnosed as livedo racemosa (LR). Several weeks later, the patient was readmitted to the clinic with an obscuration of her left visual hemifield and a bilateral HL. Ophthalmologic examination revealed extensive ischemic damage to both retinae. Now the correct diagnosis of SS was established, based on the above triad of clinical symptoms in conjunction with typical MRI and fundoscopic findings. When SS was diagnosed, the standard therapy with intravenous cyclophosphamide (IVCTX) was not instituted because of a significant risk of permanent infertility. Instead, sustained control of disease activity could be achieved with a therapeutic regime combining prednisolone, intravenous immunoglobulins (IVIG), mycophenylate mofetil (MM), and methotrexate (MTX).

**Conclusions:**

An association with LR has only been described in very few cases of SS before and further underlines the pathogenetic relationship between SS and other autoimmune diseases such as JDM. In young women with SS and the desire for a child the combination of MM and MTX may represent a reasonable alternative to IVCTX.

## Background

SS consists of the triad of encephalopathy, BRAO and HL [1,2]. The clinical presentation is highly variable. In particular, any of the above symptoms can occur first and dominate the clinical picture [2-4]. Accordingly, it has been proposed to distinguish between an encephalopathic form of SS and a recurrent BRAO subset [5]. Between the two, the former is typically more severe and shows a monophasic clinical course over a period of usually no longer than two years, during which disease activity may fluctuate widely. In MRI SS is associated with a number of rather specific signs including snowball- and spoke-like lesions on sagittal images, which correspond to microinfarcts in the centre of the corpus callosum and are deemed pathognomonic of SS [5,6]. More recently, MRI at 7 Tesla has been shown to permit a better differentiation between white matter lesions in SS and those in MS [7].

Histopathologically, SS is associated with a microangiopathy of the brain, retina and cochlea [1,3,4]. Although a detailed pathomechanism remains to be elucidated, antibody-mediated damage of endothelial cells is discussed as an important step. This includes the demonstration of activated complement components in the capillaries of SS brain biopsies [8,9]. Moreover, anti-endothelial cell antibodies (AECA) of the IgG variety have been described in the serum of patients with SS [9,10]. Although the exact specificity of these antibodies is still unknown, they have been shown to recognize a distinctive protein of 50 kDa in Western blotting which is not bound by AECA of other autoimmune diseases, including dermatomyositis (DM) [9]. More generally, the notion of an immunopathogenesis of SS is supported by the typical inflammatory constellation in cerebrospinal fluid (CSF) studies and the response to immunosuppressive treatment.

Indeed, SS is in principle amenable to immunosuppressive therapy, although in some cases disease activity has proven difficult to control [8]. Due to the absence of clinical trials, therapeutic approaches are largely based on anecdotal reports and on models of other autoimmune diseases, most notably JDM. Therapeutic regimes usually rely on a combination of corticosteroids and IVIG over an extended period of time (minimum 6 months). In more severe cases, an additional immunosuppressive therapy is recommended, preferably in the form of IVCTX pulse therapy. The combination of MM and MTX is considered an alternative, but is usually reserved for cases with less threatening disease [8].

Here, we report a case of SS that initially presented with acute encephalopathy and LR. This at first directed diagnostic considerations towards systemic lupus erythematosus (SLE) or a primary vasculitis syndrome, and the correct diagnosis of SS was established only several weeks later when the full clinical triad became manifest. Because of a risk of permanent infertility the standard immunosuppressive therapy with IVCTX was not given, but sustained control of disease activity was achieved with a combination of IVIG, MM and MTX. Our case illustrates that SS should always be considered as a differential diagnosis in a patient with acute encephalopathy, especially in the presence of additional skin manifestations, and that a therapeutic regime without CTX may be effective even in some patients with an aggressive course of the disease.

## Case presentation

A 32-year-old woman of European descent was in week 32 of her first, uncomplicated pregnancy when a subtle change in personality including a neglect of her daily duties was noticed. One week later, while on a holiday in Austria, she developed an unsteadiness of gait and slurred speech and was admitted to the hospital. Clinical examination revealed dysarthria and a hemispasticity with positive Babinski sign on the left side. While neuropsychologically still adequate on the evening of admission, she became severely confused and disoriented overnight. She did not complain of headaches at any time. Cranial MRI showed multiple small T2-intense lesions in both supra- and infratentorial locations, some of which exhibited diffusion restriction. Several lesions were found in the corpus callosum. CSF examination demonstrated a mild lymphocytic pleocytosis (13 cells/ *μ*l) and a markedly elevated protein concentration (1,800 mg/l) in the absence of oligoclonal bands. Otherwise, routine laboratory tests were normal. She delivered her child by emergency caesarean section and received antiviral therapy until negative results of virus PCR arrived. An extensive serological diagnostic did not yield evidence of viral, bacterial or protozoal infection. A diagnosis of MS was made, and a pulse of IVMP was given (5×500 mg). Several days after completion of IVMP therapy an exanthema appeared on the trunk and extremities, which was diagnosed as LR by a consultant dermatologist. A therapy with oral prednisolone was initiated, under which the exanthema resolved quickly and neurological symptoms improved gradually.

**Figure 1:**
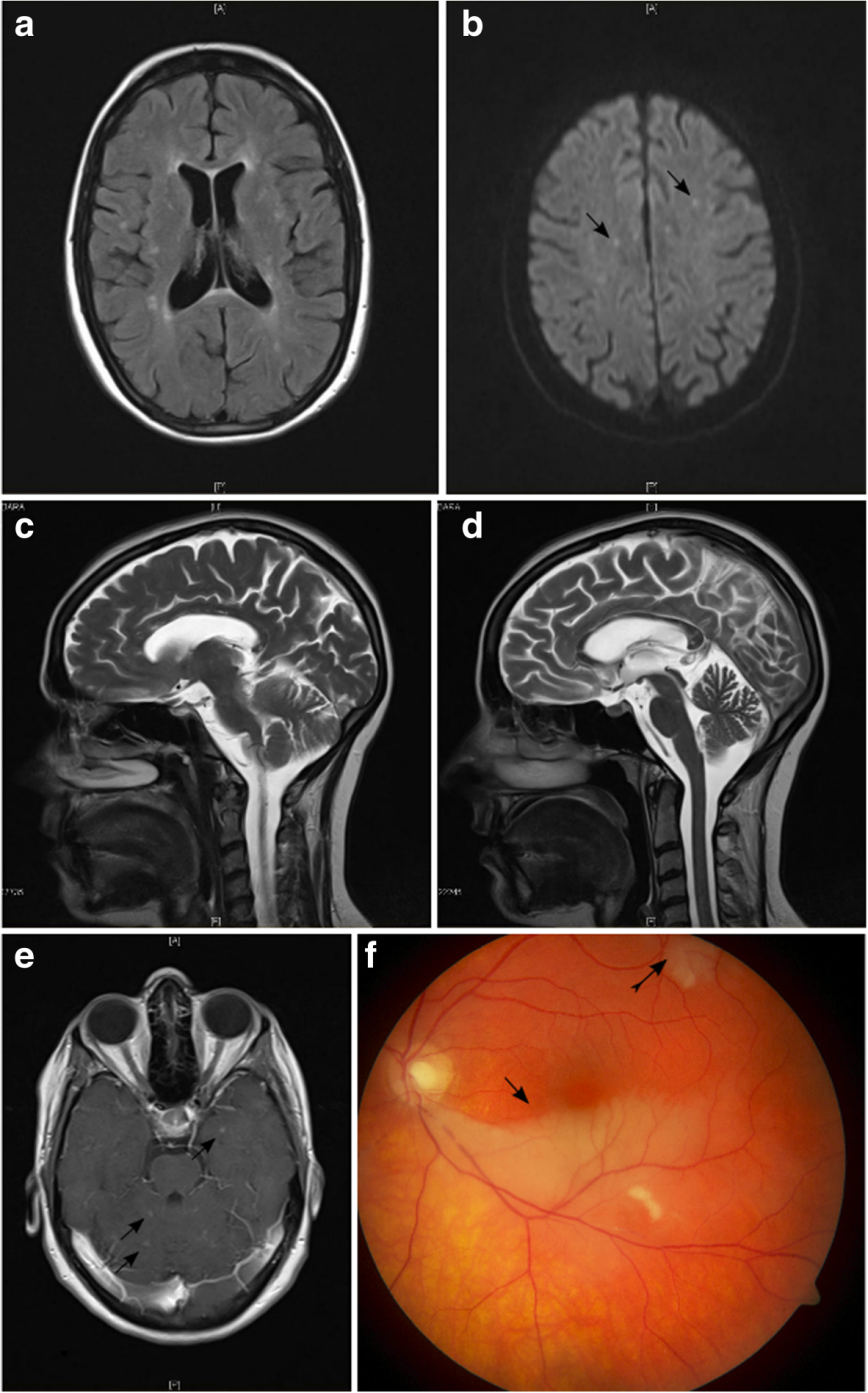
**MRI, DSA and funduscopic studies.a)** Axial fluid-attenuated inversion recovery image showing multiple small white matter lesions. **b)** Diffusion-weighted image with multiple small lesions exhibiting diffusion restriction (arrows) and correspondingly decreased ADC values (not shown). **c)** Sagittal T2 image showing centrally located corpus callosum lesions (arrow) and one lesion with spoke-like morphology (tailed arrow). **d)** Near-median sagittal T2 image demonstrating atrophy of the corpus callosum. **e)** Contrast-enhanced T1 image with multiple small contrast-enhancing lesions (arrows). **f)** Large ischemic area in the lower half of the left retina (arrow) and smaller infarct in the top left quadrant (tailed arrow).

Due to a misunderstanding, medication was not continued upon discharge from hospital. Two days later, having returned to Germany, the patient again became severely confused and apathetic and was admitted to our clinic. On neurological examination, she had an ataxic stance and gait, dysmetria, dysarthria and bilaterally hyperactive tendon reflexes. MRI demonstrated several small T2-intense lesions, some of which showed contrast enhancement and others diffusion restriction (Figure 1a-e). Some lesions were located in the centre of the corpus callosum or connected to its roof (Figure 1c). Overall, the corpus callosum appeared atrophic (Figure 1d). Since cerebral vasculitis was considered a differential diagnosis, a digital subtraction angiography was performed, but was unremarkable. Laboratory tests for anti-nuclear antibodies, anti-neutrophil cytoplasmic antibodies, anti-thyroid, anti-neuronal and paraneoplastic antibodies, viral hepatitis and human immunodeficiency virus were all negative. There was also no evidence of antiphospholipid antibodies, lupus anticoagulant or inherited thrombophilia. On admission, a bluish, net-like exanthema on the trunk and legs was noticed and diagnosed as LR. Biopsy conformation was attempted, but when a punch biopsy was performed two days after the start of another IVMP pulse (5×500 mg), the skin changes had almost vanished and the biopsy showed normal dermis. A therapy with oral prednisolone was re-initiated, and cognitive deficits and gait disorder gradually improved.

Six weeks later (when prednisolone had been tapered to 40 mg/d) the patient was readmitted to our clinic. She reported a slow worsening of her gait over the past two weeks, and two days before admission she had noticed an obscuration in her left visual hemifield. Clinical examination revealed an additional proximal weakness of the right leg and bilateral hypacusis. Ophthalmologic examination demonstrated extensive ischemic damage to both retinae with corresponding visual field deficits (Figure 1f). Audiometry showed bilateral asymmetric low-frequency sensorineural hearing loss. At this point SS was diagnosed based on the presence of the clinical triad of encephalopathy, visual disturbances and hearing loss, in conjunction with typical MRI findings (snowball lesions, callosal atrophy), CSF and fundoscopic studies. Further ophthalmologic investigations such as fluoresceine angiography (FAG) and optical coherence tomography were nor performed at this stage.

SS was diagnosed and immunosuppressive treatment initiated. This included another IVMP pulse (5×500 mg) followed by oral prednisolone (4 weeks 60 mg/d; 2 weeks 50 mg/d; 2 weeks 40 mg/d; 2 weeks 30 mg/d; 3 weeks 20 mg/d; 3 weeks 15 mg/d; 3 weeks 10 mg/d; 4 weeks 5 mg/d; 4 weeks 2.5 mg/d). IVIG were given at monthly intervals at a dose of 2 g/kg over five consecutive days for six months. In addition, a long-term therapy with MM (2000 mg/d) and MTX (15 mg weekly s.c.) was initiated. Under this regime no further relapse occurred. One year after disease onset the patient was well. Her gait and cognitive performance had markedly improved. MRI and FAG showed no evidence of disease activity.

## Conclusions

The case presented here illustrates several important aspects of SS. To start with, skin involvement in terms of LR has only been described in very few out of more than 300 patients reported in the literature so far [1,11,12]. In most cases, similar to the one presented here, the exanthema quickly resolved under immunosuppressive therapy. In one case [11], skin biopsy showed signs of complement-mediated damage to endothelial cells in upper dermis capillaries, analogous to the histopathological changes in brain biopsies of SS [9]. In combination with the case presented here, these observations strongly suggest that LR can be part of SS, and that SS is an important differential diagnosis in an encephalopathic patient with skin involvement compatible with LR.

The spectrum of differential diagnosis of LR in neurological patients has been comprehensively reviewed by Kraemer et al. [13]. In addition to Sneddon’s syndrome, the authors mention neuropsychiatric SLE and antiphospholipid antibody syndrome. To distinguish SS from these differential diagnoses can be a challenge, especially if only encephalopathy is present. In our case, the typical findings on MRI could have given an indication of the correct diagnosis SS. Specifically, lesions located in the centre or the roof of the corpus callosum are considered specific for SS because they are not found in MS or Acute Disseminated Encephalomyelitis [5]. In addition, a reduced volume of the corpus callosum as well as meningeal contrast enhancement are more frequently encountered in SS than MS [5,7]. Although it is currently less clear how well other differential diagnoses such as SLE and CNS vasculitis can be distinguished from SS by these criteria, the above MRI findings should always prompt further diagnostic procedures including FAG, which frequently reveals subclinical involvement of the retina in SS, a finding that can strongly support the diagnosis [12].

The notion of skin involvement in SS is not only relevant for clinical purposes, but may also contribute to a better understanding of the relationship between SS and other autoimmune diseases. Similarities between SS and JDM have been emphasized by some authors [8]. Both syndromes are characterized by a widely fluctuating, largely unpredictable disease activity with either a monophasic or polycyclic clinical course. Moreover, in the pathogenesis of either disease, AECA have been described [9,10]. Traditionally, it was held that, despite these commonalities, in each disease the microvasculature of a distinct set of organs was affected (i.e. brain, retina and cochlea in SS versus skin, muscle and gastrointestinal tract in DM/JDM). However, several results suggest that such a strict organ specificity does not exist. On one hand, involvement of the brain or retina has been documented in rare cases of JDM [14,15]. On the other hand, in SS a histological involvement of skeletal muscle can be observed [3,4], and the case presented here in combination with the report by Turc et al. demonstrate a skin involvement with similar features as in DM. Altogether these findings further underline a pathogenetic relationship between SS and DM/JDM.

The case presented here further exemplifies a therapeutic dilemma that may be commonly encountered in SS. On one hand, in the case of threatening disease activity, prompt and sustained aggressive immunosupression is warranted to prevent severe sequelae such as dementia, vision loss and hearing loss. On the other hand, every long-term immunosuppressive therapy is likely to have significant adverse effects, and for this reason any unnecessarily aggressive treatment must be avoided. In the context of autoimmune disease, IVCTX pulse therapy causes premature ovarian failure in a relatively high proportion of premenopausal women, especially in those older than 30 years [16,17]. Various strategies of fertility preservation treatment have been evaluated, but all are associated with a significant residual risk of infertility. In contrast, immunosuppressive therapy with MM and MTX is not expected to have long-term adverse effects on fertility once therapy has been completed [18].

Given the overall severe clinical presentation in our patient, including a tendency to relapse when tapering prednisolone, most physicians would probably have started an IVCTX pulse therapy when the diagnosis of SS was eventually made. However, this was not wanted by the patient and her family, primarily because they intended to have further children. Instead, a long-term immunosuppressive therapy with MM and MTX was initiated, and at the same time an IVIG pulse therapy was newly started. Interestingly, from this point on the clinical course was much more benign, and even relatively quick tapering of prednisolone because of a severe Cushing’s syndrome was tolerated well. Although it is impossible to know whether this reflects the natural course of the disease or a therapeutic effect of MM/MTX and/or IVIG, our case suggests that even in patients with an initially threatening presentation of SS, disease activity can sometimes be controlled by immunosuppresive treatment that does not rely upon IVCTX. Given that SS often occurs in young women before the completion of family planning, the value of such treatment options should be systematically compared to the standard therapy including IVCTX.

## Consent

Written informed consent was obtained from the patient for publication of this Case report and any accompanying images. A copy of the written consent is available for review by the Editor of this journal.

## Abbreviations

AECA: Anti-endothelial cell antibodies; BRAO: Branch retinal artery occlusions; CSF: Cerebrospinal fluid; DM: Dermatomyositis; FAG: Fluoresceine angiography; HL: Hearing loss; IVCTX: Intravenous cyclophosphamide; IVIG: Intravenous immunoglobulins; IVMP: Intravenous methylprednisolone; JDM: Juvenile dermatomyositis; LR: Livedo racemosa; MM: Mycophenylate mofetil; MRI: Magnetic resonance imaging; MS: Multiple sclerosis; MTX: Methotrexate; SLE: Systemic lupus erythematosus; SS: Susac’s Syndrome.

## Competing interests

The authors declare that they have no competing interests.

## Authors’ contributions

ME, HL, IK and MD saw the patient, diagnosed SS and conducted the immunosuppressive therapy; BLK performed the ophthalmologic examination; HR and TL performed neuroradiologic studies; and MH gave advise with respect to immunosuppressive therapy. ME and MD wrote the manuscript with contributions from all other authors. All authors read and approved the final version of the manuscript.

## Pre-publication history

The pre-publication history for this paper can be accessed here:

http://www.biomedcentral.com/1471-2377/13/185/prepub

## References

[B1] Susac JO Hardman JM Selhorst JB Microangiopathy of the brain and retina Neurology 1979 29 3 313 316 10.1212/WNL.29.3.313 571975

[B2] Susac JO Susac’s syndrome: the triad of microangiopathy of the brain and retina with hearing loss in young women Neurology 1994 44 4 591 593 10.1212/WNL.44.4.591 8164809

[B3] Petty GW Engel AG Younge BR Duffy J Yanagihara T Lucchinetti CF Bartleson JD Parisi JE Kasperbauer JL Rodriguez M Retinocochleocerebral vasculopathy Medicine (Baltimore) 1998 77 12 40 10.1097/00005792-199801000-00003 9465861

[B4] O’Halloran HS Pearson PA Lee WB Susac JO Berger JR Microangiopathy of the brain, retina, and cochlea (Susac syndrome). A report of five cases and a review of the literature Ophthalmology 1998 105 6 1038 1044 http://dx.doi.org/10.1016/S0161-6420(98)96005-5 10.1016/S0161-6420(98)96005-5 9627654

[B5] Rennebohm R Susac JO Egan RA Daroff RB Susac’s syndrome–update J Neurol Sci 2010 299 1–2 86 91 http://dx.doi.org/10.1016/j.jns.2010.08.032 20855088 10.1016/j.jns.2010.08.032

[B6] Susac JO Murtagh FR Egan RA Berger JR Bakshi R Lincoff N Gean AD Galetta SL Fox RJ Costello FE Lee AG Clark J Layzer RB Daroff RB MRI findings in Susac’s syndrome Neurology 2003 61 12 1783 1787 10.1212/01.WNL.0000103880.29693.48 14694047

[B7] Wuerfel J Sinnecker T Ringelstein EB Jarius S Schwindt W Niendorf T Paul F Kleffner I Dörr J Lesion morphology at 7 Tesla MRI differentiates Susac syndrome from multiple sclerosis Mult Scler 2012 18 11 1592 1599 http://dx.doi.org/10.1177/1352458512441270 10.1177/1352458512441270 22711711

[B8] Rennebohm RM Susac JO Treatment of Susac’s syndrome J Neurol Sci 2007 257 1–2 215 220 http://dx.doi.org/10.1016/j.jns.2007.01.031 17324441 10.1016/j.jns.2007.01.031

[B9] Magro CM Poe JC Lubow M Susac JO Susac syndrome: an organ-specific autoimmune endotheliopathy syndrome associated with anti-endothelial cell antibodies Am J Clin Pathol 2011 136 6 903 912 http://dx.doi.org/10.1309/AJCPERI7LC4VNFYK 10.1309/AJCPERI7LC4VNFYK 22095376

[B10] Jarius S Neumayer B Wandinger KP Hartmann M Wildemann B Anti-endothelial serum antibodies in a patient with Susac’s syndrome J Neurol Sci 2009 285 1–2 259 261 http://dx.doi.org/10.1016/j.jns.2009.07.002 19643446 10.1016/j.jns.2009.07.002

[B11] Turc G Monnet D Dupin N Beuvon F Guiraud V Amor MB Touzé E Skin involvement in Susac’s syndrome J Neurol Sci 2011 305 1–2 152 155 http://dx.doi.org/10.1016/j.jns.2011.03.001 21440909 10.1016/j.jns.2011.03.001

[B12] Dörr J Krautwald S Wildemann B Jarius S Ringelstein M Duning T Aktas O Ringelstein EB Paul F Kleffner I Characteristics of Susac syndrome: a review of all reported cases Nat Rev Neurol 2013 9 6 307 316 http://dx.doi.org/10.1038/nrneurol.2013.82 10.1038/nrneurol.2013.82 23628737

[B13] Kraemer M Linden D Berlit P The spectrum of differential diagnosis in neurological patients with livedo reticularis and livedo racemosa. A literature review J Neurol 2005 252 10 1155 1166 http://dx.doi.org/10.1007/s00415-005-0967-9 10.1007/s00415-005-0967-9 16133722

[B14] Jimenez C Rowe PC Keene D Cardiac and central nervous system vasculitis in a child with dermatomyositis J Child Neurol 1994 9 3 297 300 10.1177/088307389400900315 7930410

[B15] Elst EF Kamphuis SSM Prakken BJ Wulffraat NM van der Net J Peters ACB Kuis W Case report: severe central nervous system involvement in juvenile dermatomyositis J Rheumatol 2003 30 9 2059 2063 12966616

[B16] Boumpas DT Austin HA Vaughan EM Yarboro CH Klippel JH Balow JE Risk for sustained amenorrhea in patients with systemic lupus erythematosus receiving intermittent pulse cyclophosphamide therapy Ann Intern Med 1993 119 5 366 369 10.7326/0003-4819-119-5-199309010-00003 8338289

[B17] Ioannidis JPA Katsifis GE Tzioufas AG Moutsopoulos HM Predictors of sustained amenorrhea from pulsed intravenous cyclophosphamide in premenopausal women with systemic lupus erythematosus J Rheumatol 2002 29 10 2129 2135 12375322

[B18] Henes M Henes JC Neunhoeffer E Wolff MV Schmalzing M Kötter I Lawrenz B Fertility preservation methods in young women with systemic lupus erythematosus prior to cytotoxic therapy: experiences from the FertiPROTEKT network Lupus 2012 21 9 953 958 http://dx.doi.org/10.1177/0961203312442753 10.1177/0961203312442753 22438026

